# GoBot Go! Using a Custom Assistive Robot to Promote Physical Activity in Children

**DOI:** 10.1109/JTEHM.2024.3446511

**Published:** 2024-08-20

**Authors:** Rafael Morales Mayoral, Ameer Helmi, Samuel W. Logan, Naomi T. Fitter

**Affiliations:** Collaborative Robotics and Intelligent Systems (CoRIS) Institute, Oregon State University 2694 Corvallis OR 97331 USA; Disability and Mobility Do-it-Yourself Co-OpOregon State University 2694 Corvallis OR 97331 USA

**Keywords:** Assistive robotics, physical activity promotion, child-robot interaction

## Abstract

Children worldwide are becoming increasingly inactive, leading to significant wellness challenges. Initial findings from our research team indicate that robots could potentially provide a more effective approach (compared to other age-appropriate toys) for encouraging physical activity in children. However, the basis of this past work relied on either interactions with groups of children (making it challenging to isolate specific factors that influenced activity levels) or a preliminary version of results of the present study (which centered on just a single more exploratory method for assessing child movement). This paper delves into more controlled interactions involving a single robot and a child participant, while also considering observations over an extended period to mitigate the influence of novelty on the study outcomes. We discuss the outcomes of a two-month-long deployment, during which $N=8$ participants engaged with our custom robot, GoBot, in weekly sessions. During each session, the children experienced three different conditions: a teleoperated robot mode, a semi-autonomous robot mode, and a control condition in which the robot was present but inactive. Compared to our past related work, the results expanded our findings by confirming with greater clout (based on multiple data streams, including one more robust measure compared to the past related work) that children tended to be more physically active when the robot was active, and interestingly, there were no significant differences between the teleoperated and semi-autonomous modes in terms of our study measures. These insights can inform future applications of assistive robots in child motor interventions, including the guiding of appropriate levels of autonomy for these systems. This study demonstrates that incorporating robotic systems into play environments can boost physical activity in young children, indicating potential implementation in settings crafted to enhance children’s physical movement.

## I. Introduction

Physical activity plays an essential role in fostering young children’s overall health, contributing positively to not only cognitive, social, and motor development [1], [2], [3], but also improving later psychosocial and cardiometabolic health [4] and the construction of a foundation for healthy habits. Unfortunately, research indicates that a significant number of children are not meeting the recommended physical activity level guidelines [5], a fact that is contributing to high current levels of childhood obesity and other negative health outcomes [6]. While toys that motivate crawling and assist with children’s walking are widespread, there is a notable scarcity of toys designed to motivate young children to be active and explore their environments once they are ambulatory. By introducing robots as motivators for physical activity, we can offer attention-grabbing features (e.g., lights and sounds [7], [8]) while making an adaptable system that is customizable across users. Past research has also shown that robots can be more motivational and peer-like than other types of technology [9], [10], which can lead to potential positive outcomes such as our work’s envisioned robot-mediated physical activity promotion for young children.

Assistive robotics, the study of how robots can support people in situations from health interventions to education [11], offers one potentially groundbreaking solution for addressing the sedentary behavior epidemic by motivating child movement and exploration. A notable example of this potential is demonstrated in a single-session pilot study that utilized a Sphero robot to encourage infants to explore an environment [12]. To encourage young children to engage in physical activity and explore, our team previously designed and built an assistive mobile robot with self-propulsion abilities and built-in toy-inspired features (i.e., lights, sounds, and bubbles) [13]. This new paper covers an evaluation of this robot with a larger number of users and over a longer timescale.

**FIGURE 1.**
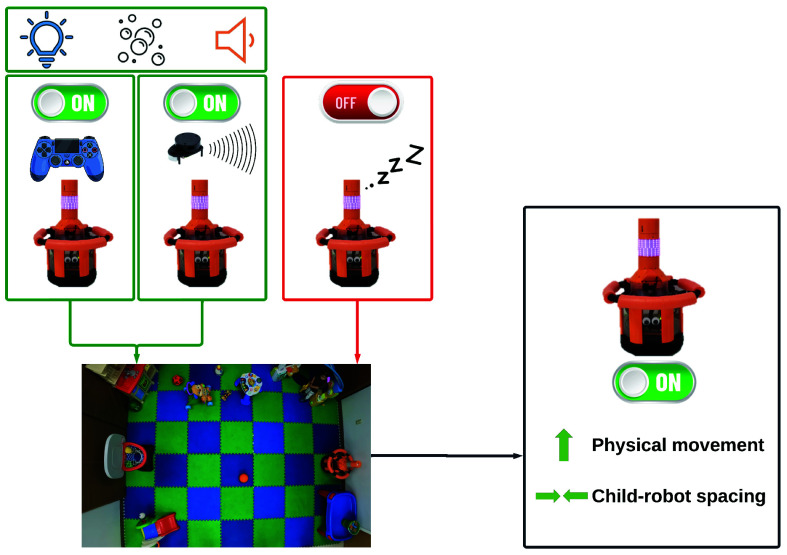
Overview of our study design and key results. The lower right black rectangle illustrates the positive results of the robot on child users.

The central research objectives behind this work were to assess *whether a mobile assistive robotic system can promote and encourage children with typical development to move and how this intervention’s success changes over time when incorporating different methods to track movement.* We approached this topic by studying child-robot interaction in a lab setting over multiple months of interaction. In this paper, Section II discusses how robots possess a unique peer-like presence that may be unique (compared to other technologies) for encouraging healthy behaviors. Our assistive robot, GoBot, as described in Section III and shown in Fig. 1, interacted with eight child participants over two months of study sessions (Section IV). The results in Section V hint that the presence of an active robot in the play space is beneficial, whether the robot is directly teleoperated or semi-autonomous. Section VI discusses main insights and important context for the work. The work contributes 1) empirical findings within the emerging field of mobile assistive robotics and 2) a semi-autonomous control strategy capable of eliciting the same child motion levels as observed during direct human teleoperation.

## II. Related Work

Related work in the promotion of physical activity, assistive robotics, and novelty in human-robot interaction informed our longitudinal study design.

*Promoting Physical Activity:* Approaches from broad national initiatives to commercial products have been developed with the goal of increasing physical activity levels for children. The “Let’s Move!” program was developed with former First Lady Michelle Obama and focused on promoting physical activity for children, providing parents with tools for better food choices, and increasing awareness of the child obesity epidemic in the United States [14]. While the program showed some impact in terms of obesity rates for very young children, the overall prevalence of childhood obesity has not significantly diminished since its introduction [15]. Technological solutions for encouraging physical activity include video games (e.g., Ring Fit Adventure [16]) and smartphone applications (e.g., the applications mentioned in [17]). These types of technologies have shown some efficacy in promoting physical activity, especially in short-term use, but require further longitudinal study to understand their influence beyond the point of novelty [17]. Assistive robots like GoBot may offer an engagement advantage compared to other tools for physical activity promotion due to people’s tendency to view robots as more peer-like and influential than non-embodied technologies such as phones or computers [9]. We designed our robot to facilitate developmentally appropriate interactions, which we thought might effectively encourage child motion over repeated sessions.

*Assistive Robots for Physical Activity:* Assistive robots for physical activity promotion have been mainly targeted towards older adults, with occasional instances of work focused on young children. In the older adult space, Gorer et al. used a NAO robot as an exercise coach [18], and robots have supported rehabilitation activities for individuals (often older adults) after a stroke [19], [20]. In work for promoting child activity, assistive robots have shown initial promise for supporting the motor development of children with cerebral palsy [21] and autism spectrum disorder [22]. NAO and Dash robots were used in tandem in past work to encourage a child with Down syndrome to perform motor activities such as crawling up a ramp [23]. For more general child populations, the “Cratus” robot encouraged children to vigorously move the robot and themselves while playing a game in other related work [24]. Our own preliminary studies with GoBot showed that the robot could encourage standing and engagement while the robot was active [25]. The small sample sizes and short study durations of the past efforts warrant further follow-up research; our present work aimed to address these gaps.

*Novelty in Human-Robot Interaction:* Human interactions with a robot or other technologies for the first time often shows a novelty effect which changes after repeated interactions [26]. For example, users might become less interested in a technology as they habituate to it. Accordingly, it is imperative to perform longer-term empirical studies to understand the impact of robots, but most longitudinal studies to date have been with older adults [27] or in applications outside of physical activity promotion, such as therapy [28] or education [29]. Kanda et al. suggest that two weeks are needed for the robot impact to show up in a human-robot interaction study [30], while Sung et al. suggest that two months are needed to get past the point of novelty in a human-robot interaction study [31]. Thus, we conducted our study over a two-month timeline to begin to understand the long-term effects of GoBot in promoting physical activity for young children.

## III. System Design

This section describes the GoBot robotic system and key operating mode information that is needed for understanding our study design and results.

### A. Robotic System

GoBot, the assistive robot used in this study, is a custom robotic system designed in collaboration with the Oregon State Disability and Mobility Do-it-Yourself Co-Op, as pictured in Fig. 1. GoBot’s components (i.e., a mobile TurtleBot2 base with onboard Raspberry Pi 4 processor, that can be directly teleoperated with a PlayStation DualShock4 [PS4] controller or execute autonomous LiDAR-based routines) and three rewards (i.e., custom lights, sounds, and bubbles) are explained in our previous work [32]. For the safety of the robot and of users, GoBot is surrounded by a foam-padded roll cage that cushions any impacts with the environment. Additional safety measures include covers for the robot’s onboard user interface and an enclosure around the TurtleBot base that prevents children from deactivating the robot or reaching any robot wiring. GoBot’s design process is described in our previous work [13].

### B. Robot Operating Modes

**FIGURE 2.**
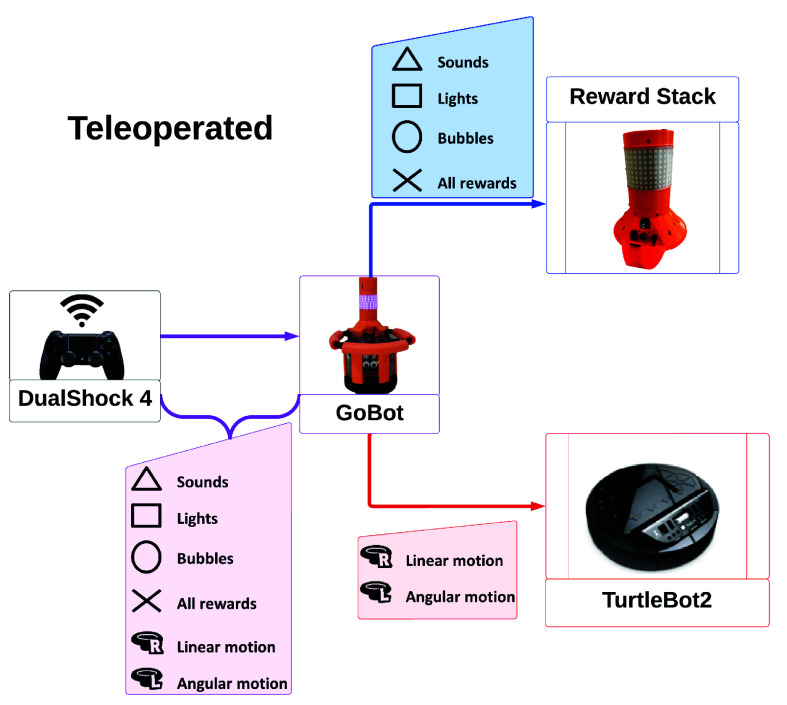
Schematic flow of the teleoperated mode, which operates fully manually. The robot movements are activated using the joysticks, and rewards are activated using the buttons.

**FIGURE 3.**
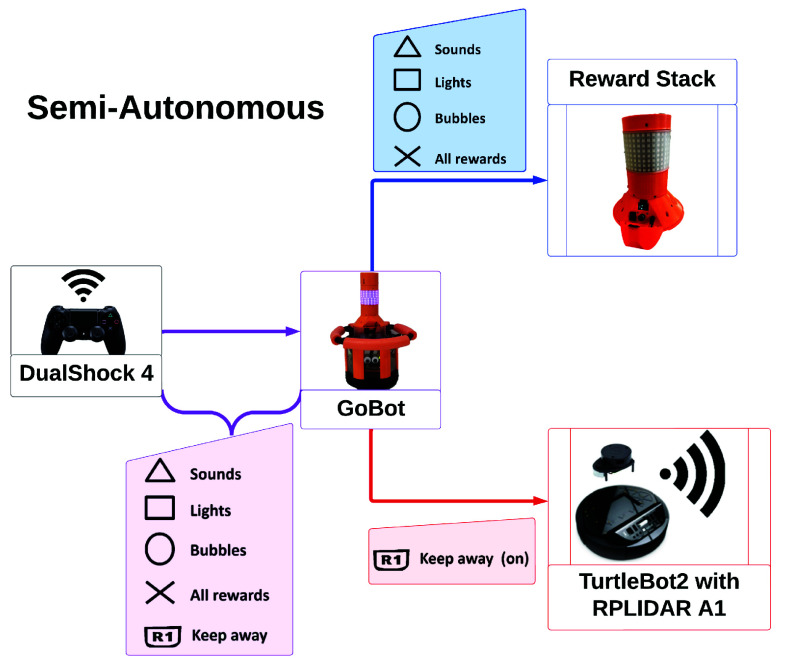
Schematic flow of the semi-autonomous mode, which can be turned on/off by pressing the R1 trigger button on the controller. In this mode, GoBot uses LiDAR sensing to autonomously perform a keep away routine, while a human operator manually activates rewards.

GoBot was designed to be user-friendly, enabling individuals with little-to-no robotics experience (such as clinicians, kinesiologists, and parents) to operate GoBot with minimal training. In the present work, GoBot operated in two modes: *teleoperated* and *semi-autonomous*. A diagram displaying the operating modes appears in Fig. 2 and 3. The use of the operation modes in the child-robot interaction study conditions is further explained in Section IV.

In the *teleoperated mode*, GoBot’s base motion and reward deployment were fully controlled by a human operator via the left joystick for linear movement, right joystick for angular movement, and x, square, circle, and triangle for rewards on the PS4 controller. The mapping for the actions can be seen in Fig. 2. The goal of the operator was to entice the child to follow GoBot by performing four different patterns of movement (i.e., moving in a circle, square, X, or triangle) across the play area while deploying rewards. Each reward was activated at least once per session, but otherwise, the operator freely combined rewards as deemed appropriate when the child was within 1 ft (30.5 cm) of the robot (i.e., to reward interaction) or more than 2 ft (61.0 cm) away from the robot (i.e., to encourage re-engagement).

In *semi-autonomous mode*, GoBot executed a keep away algorithm, which is explained in our previous work [32]. Briefly, in this mode, the robot flees the nearest object.

## IV. Methodology

To investigate GoBot’s effect on child physical activity over time, we conducted a two-month-long child-robot interaction study. Our university ethics board approved this study under protocol $\#$IRB-2020-0723.

### A. Study Design

To assess the impact of different conditions on promoting child movement during the study sessions, a within-subjects experiment was conducted. We compared the effects of the following three conditions:

• *Control condition* (10 minutes per session): GoBot was present during the play session but was not active. The children could still interact with GoBot (i.e., touching, pushing, pulling) the passive robot.
• *Teleoperated condition* (Experimental condition 1; 5 minutes per session): GoBot was fully teleoperated by a research team member, using the protocol more fully described in Section III-B.
• *Semi-autonomous condition* (Experimental condition 2; 5 minutes per session): GoBot ran in the semi-autonomous mode, as fully described in Section III-B. In short, the base motion was autonomous, rewards were triggered manually, and autonomous behavior was interruptable.

In all three conditions, the child had the freedom to engage with a variety of developmentally appropriate toys within the designated play space. A modified Latin squares method was employed to maintain a balanced order of conditions across the user group.

To gain a longitudinal perspective on participant experiences, the study spanned a duration of *two months*. Participants attended *eight weekly sessions*, where each session followed a pre-assigned sequence of the three aforementioned conditions. This approach allowed for a comprehensive examination of the participants’ experiences over an extended period.

### B. Participants

Eight participants took part in the study (5 male, 3 female). We recruited participants through local daycares and farmers’ markets. Participant ages ranged from 2.01 to 3.35 years old ( $M = 2.52$ and $SD = 0.50$). All recruited children were typically developing, and three had previous experience with other robots (specific robots not recorded).

### C. Measures

We used a mixed-methods approach and collected two types of data during our study: behavioral and self-reported. The behavioral data included measurements from wearable sensors, as well as footage captured by an overhead video camera, documenting each play session. The self-reported data comprised of parent responses to surveys.

#### 1) Behavioral Measures

Accelerometer and gyroscope data was recorded at 100 Hz using three GT9X ActiGraph sensors, which the child wore on the wrist, ankle, and hip. A GoPro Hero Black 10 camera running at 30 Hz was used to record overhead footage. We also used a GoPro Hero Black 7 running at 30 Hz to record a side view of the play space. We captured a front-facing view of the session using a Canon camera. These recordings were used to capture information on child motion levels and proximity to the robot.

#### 2) Self-Reported Measures

The parents of the participants involved in the study completed surveys about general and study-specific experiences with robots at the beginning of the study, as well as after each session and at the end of the study. In the *pre-study survey*, we used the Likert-type standard questions of the Negative Attitudes towards Robots Scale (NARS) [33] and the Trust Perception Scale-HRI [34] to gauge pre-existing participant perceptions of robots. Demographic questions captured information about participant age, gender, and development. Finally, free-response survey questions asked parents about experiences with robots and thoughts on robot usefulness. The *post-session survey* included questions about child engagement with the robot and perceptions of GoBot. Custom Likert-type questions in this survey asked the parent to rate child engagement with GoBot, general perception of GoBot, and belief in robot usefulness for child well-being on a 7-point Likert-type scale from Strongly Disagree (1) to Strongly Agree (7). Parents also completed free-response questions about perceptions of the robot and child-robot interactions during each session. In the *post-study survey*, the same NARS and trust perception questions were asked as in the pre-study survey. Free-response questions asked parents about perceptions of GoBot and child interactions with the robot, as well as ideas for system use and changes to the system.

### D. Procedure

Prior to commencing the study, parental informed consent was obtained. At the start of the first session, before the initiation of play, parents completed the pre-study survey and a demographic survey. During each session, the child wore the three ActiGraph sensors, positioned on the right ankle, right wrist, and hip.

**FIGURE 4.**
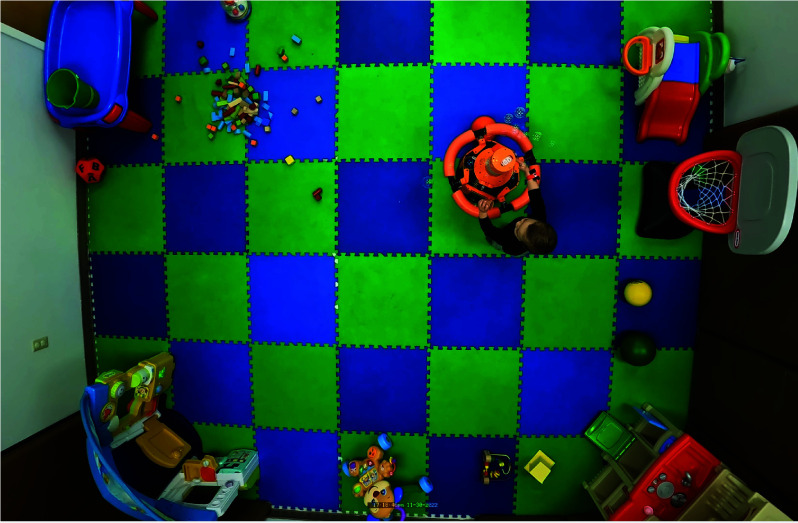
Overhead view of the play environment showing a participant interacting with the robot.

In each session, the three conditions (i.e., control, teleoperated, and semi-autonomous) occurred in the pre-assigned order. During the session, the child was in a play area with a consistent and developmentally appropriate assortment of toys, which can be seen in Fig. 4. Parents were also present in the study space. Children played freely in the space during each session.

After each play session, the sensors were removed from the child and the parents completed the post-session survey and were compensated ${\$}$25 for their participation. The full study lasted eight sessions with each session lasting about 25 minutes. After the last session, parents completed the post-study survey.

### E. Hypotheses

In this work, we tested four hypotheses:

H1: The children will move more during the experimental conditions (i.e., teleoperated and semi-autonomous) compared to the control condition. This idea is supported by past single-session work on robot-mediated physical activity promotion for children [24]; our efforts assess the same idea in a longer-term context.
H2: Child physical activity levels will be similar between the two experimental conditions. This hypothesis is based on observations from our own exploratory pilot sessions; both experimental modes appeared to be promising for encouraging movement.
H3: The effectiveness of the robot for motivating motion will decrease over time. This hypothesis is based on related work on the novelty effect (e.g., [26]), which typically shows a decline in interest in new technologies over the course of habituation.
H4: Proximity between the child and robot will be closer for both experimental conditions compared to baseline. This hypothesis is based on informal observations during pilot sessions.

### F. Analysis

We analyzed the data from the ankle-mounted ActiGraph sensor (as this location specifically reflects repetitive walking and running patterns well [35]), overhead GoPro camera, and self-reported survey responses focused on perceptions of the child’s interaction with GoBot and general perceptions of robots.

For all session-wise objective data, to obtain comparable values across conditions, we normalized the measured outcomes by the duration of the condition. We tested for significant differences between conditions and across sessions using a two-way repeated measures analysis of variance (rANOVA) test. The rANOVA used an $\alpha =0.05$ significance level and were conducted using jamovi 2.3.18 [36], [37]. We used Tukey’s HSD test for pairwise comparisons in the case of significant main effects. We report effect sizes using $\eta ^{2}$, where $\eta ^{2} =0.01$ is considered a small effect, $\eta ^{2} =0.04$ is a medium effect, and $\eta ^{2} =0.09$ is a large effect [38].

*ActiGraph Data:* We first extracted the accelerometer and gyroscope data from the ActiGraph sensor using the *ActiLife* version 6.13.4 software. This data was evaluated for ankle movement counts using the algorithm presented in [39]. Based on this algorithm, we used each participant’s raw ankle sensor recordings to calculate the root mean square (RMS) acceleration and angular velocity, and then computed the specific thresholds for acceleration and velocity of each participant from the individual user datasets. To begin this computation, detrending was performed utilizing the median. Data points falling outside the rejection range of a=[−1.02, 1.32] m/s^2^ and below the value of w=[0.32] rad/s were then excluded. Following this step, a moving average filter with a window size of 0.5 seconds was applied to smooth the data, reducing noise. Next, we identified peaks exceeding 1.0 m/s^2^ for acceleration and 0.1 rad/s for angular data, which helped to distinguish important changes in the dataset. Finally, each participant’s unique threshold was set by taking the mean of the local maxima and subtracting half the standard deviation. A so-called vigorous movement started when both velocity and acceleration exceeded their respective thresholds and stopped when acceleration and velocity returned to a level below the threshold. An example of the ankle movement data can be seen in our previous work [32]. We analyzed only the ankle sensor recordings since we were most interested in walking movement in the present study.

*Overhead Video Tracking:* For each session, we utilized the overhead video captured by the GoPro camera and employed a specialized region-of-interest (ROI) tracker, OverTrack, to estimate the children’s overground movement during each session. OverTrack is publicly available for use [40] and has been validated for use as a tool for post hoc positional analysis in [41]. For every video, the first step entailed a researcher manually creating bounding boxes around the child, the robot, and the play environment. The researcher would then supply the tool with a reference measurement from the environment using one of the floor foam mats, which measure $2\times 2$ feet ( $61.0\times 61.0$cm). When the tracker lost track or sight of a region of interest, the researcher would redraw boxes as needed. The play environment is shown in Fig. 4. The ROI tracker outputted the centroids of the bounding boxes for both the robot and the child in each video frame. To determine the overall extent of the child’s movement during each session, we computed the cumulative change in the child’s centroid location between consecutive frames, excluding any positional changes exceeding 0.5 feet (15.2cm; unlikely considering the maximum speed of the child ambulation [42]), as well as changes smaller than 0.06 feet (1.8cm; likely to be noise). By implementing a different (and less exploratory) method to evaluate child movement, we were able to collect more information to validate the actual child physical movement. This reading, coupled with the ActiGraph data, help us further understand how effective GoBot is to promote physical movement.

*Child-Robot Spacing:* Utilizing the same data outputted by OverTrack, we calculated the spacing between the child and robot during sessions using the Euclidean distance between the centroids of the bounding boxes. We report the mean and standard deviation of this value across sessions and conditions.

*Survey Responses:* We used the session-wise survey data to understand engagement and well-being perceptions and the pre- and post-study surveys to compare attitudes towards robots over the course of the study to further understand the parents’ attitude towards GoBot’s engagement of their child by exploring social and emotional aspects of the interaction. The engagement and well-being self-reports were collected only once per session and thus could not be used to compare across condition experiences; the descriptive statistics of these ratings mainly helped to provide a rough understanding of perceived experiences. To compare the pre- and post-study NARS and trust ratings, we performed one-way rANOVA tests with an $\alpha =0.05$ significance level.

## V. Results

All participants successfully completed the full eight sessions of the study protocol. Recording errors occurred for the ActiGraph data during two sessions (one session for each of two participants). Recording errors also occurred in the overhead camera footage for one session with Participant 2 (for all conditions) and one session with Participant 4 (for the experimental conditions). All other sensor and survey data was successfully captured. The results for the ActiGraph recordings, overhead tracking, child-robot spacing, and survey data are presented below.

**FIGURE 5.**
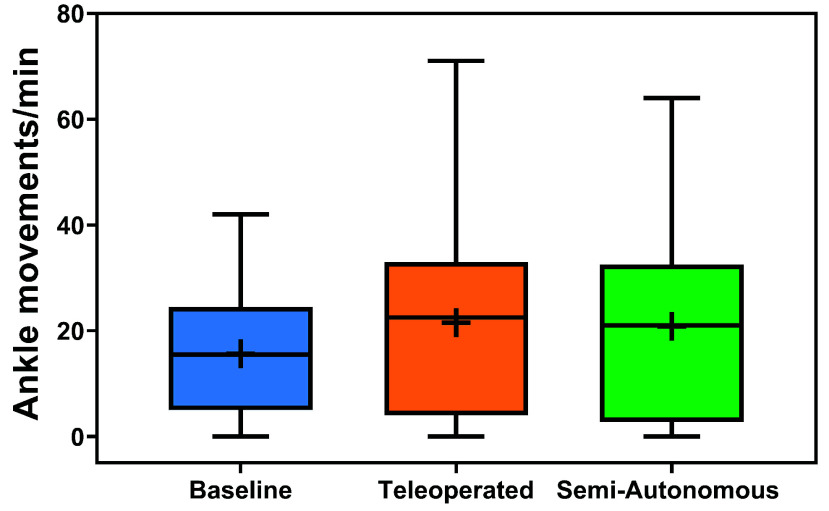
Distributions of normalized ankle movements per minute across conditions. Boxplots include boxes from the 25th to the 75th percentiles, center lines with a circle marker for medians, asterisks for means, whiskers up to 1.5 times the interquartile range.

**FIGURE 6.**
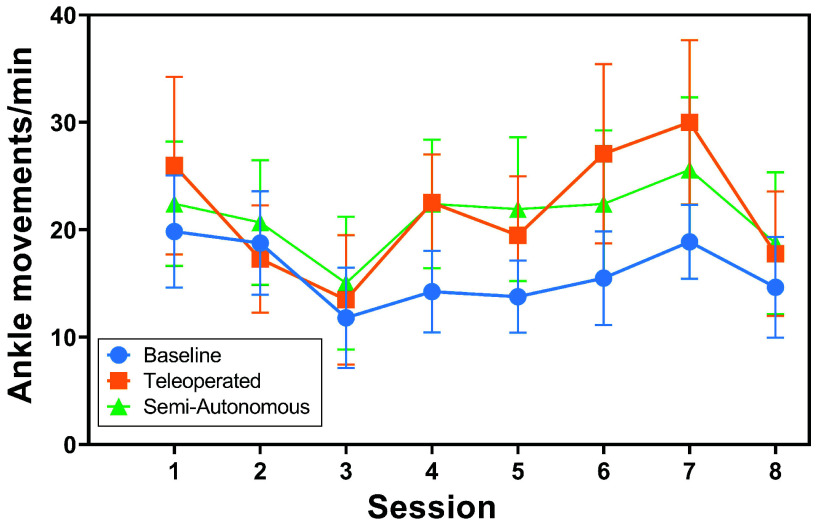
Ankle movements per minute over study session. Markers show the mean and error bars show standard error.

*ActiGraph Results:* The distributions of normalized ankle movements across conditions and over time are illustrated in Figs. 5 and 6. The results of the two-way rANOVA across conditions and sessions showed a significant main effect for conditions (*F*(2,10) =4.29, $p =0.045$, $\eta ^{2} =0.028$). However, no pairwise differences were significant after post hoc comparisons with Tukey’s HSD. There was no significant main effect across sessions ( $p =0.804$). The average ankle motion rates tended to be higher for both experimental conditions (compared to the control) for all sessions but one. Specifically, compared to the control, the average ankle movement rates were higher for the teleoperated condition during seven sessions and were higher for the semi-autonomous condition during all eight sessions.

**FIGURE 7.**
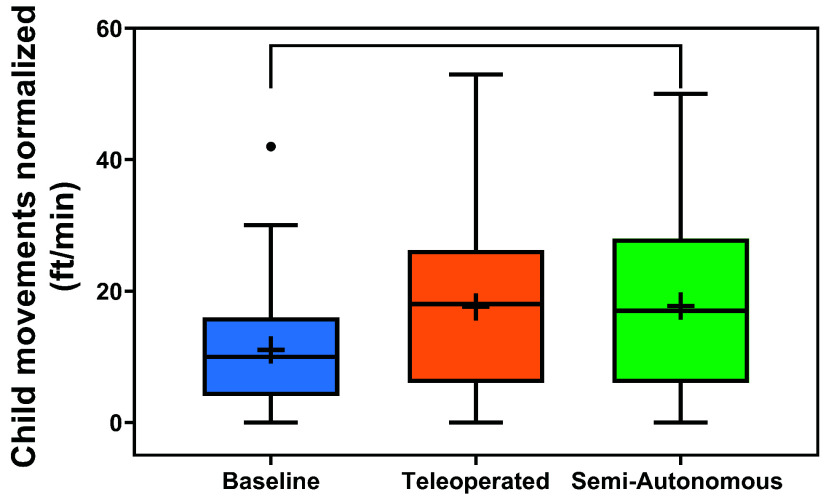
Distributions of normalized child total movement across conditions. Boxplots include boxes from the 25th to the 75th percentiles, center lines with a circle marker for medians, asterisks for means, brackets for significance, and whiskers up to 1.5 times the interquartile range.

**FIGURE 8.**
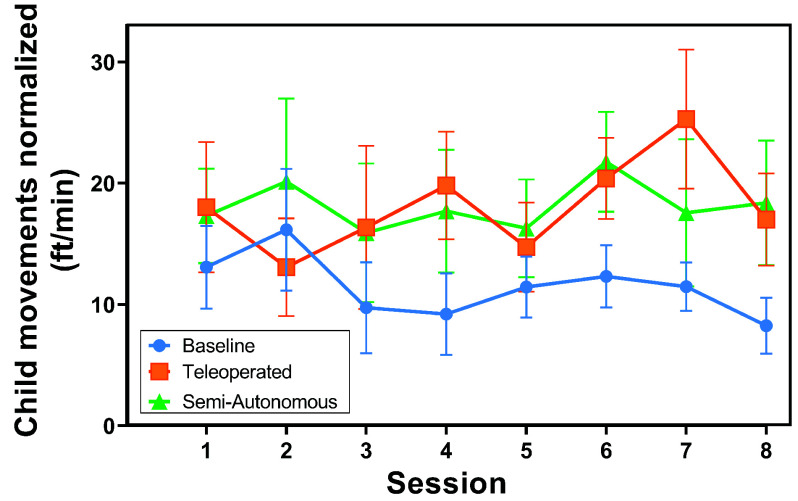
Normalized child overground movement over study session. The markers show the mean, and the error bars illustrate the standard error.

*Overhead Video Results:* The distributions of normalized child movement across conditions and over time are illustrated in Figs. 7 and 8. The results of the two-way rANOVA across conditions and sessions showed a significant main effect for conditions (*F*(2,10) =4.10, $p =0.050$, $\eta ^{2} =0.071$). Pairwise testing showed significantly higher movement rates for the semi-autonomous condition than the control condition ( $p =0.048$). There was no significant main effect across sessions ( $p =0.701$). The average child motion rates tended to be consistently higher in both experimental conditions compared to the control across most sessions. More precisely, in comparison to the control, the child movement rates were elevated during seven sessions for the teleoperated condition and during all eight sessions for the semi-autonomous condition.

**FIGURE 9.**
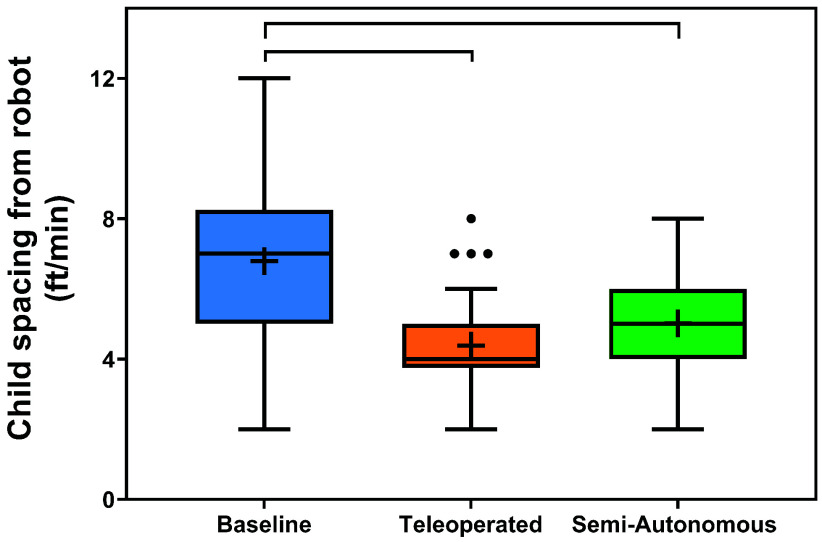
Distributions of child distance from the robot per minute across conditions. Boxplots include boxes from the 25th to the 75th percentiles, center lines with a circle marker for medians, asterisks for means, brackets for significance, and whiskers up to 1.5 times the interquartile range.

**FIGURE 10.**
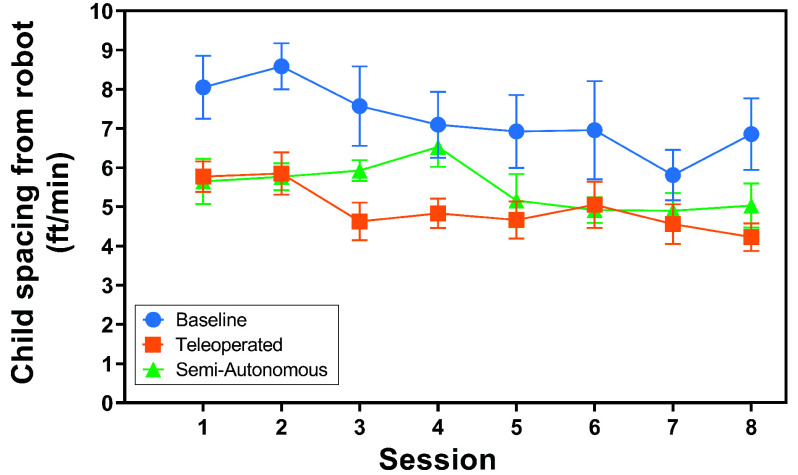
Child distance from robot per minute over study session. Here, a lower value represents the child being closer to the robot. The markers show the mean, and the error bars illustrate the standard error.

*Child-Robot Spacing Results:* The distributions of the children’s spacing from the robot across conditions and over time are illustrated in Figs. 9 and 10. The results of the two-way rANOVA across conditions and sessions showed a significant main effect for conditions (F(2,6) =15.68, $p =0.004$, $\eta ^{2} =0.151$). Pairwise significance showed that compared to the control condition, the teleoperated condition ( $p =0.036$) and semi-autonomous condition ( $p =0.033$) both yielded significantly closer child-robot spacing. The young children were closer to the robot when the robot was active.

**FIGURE 11.**
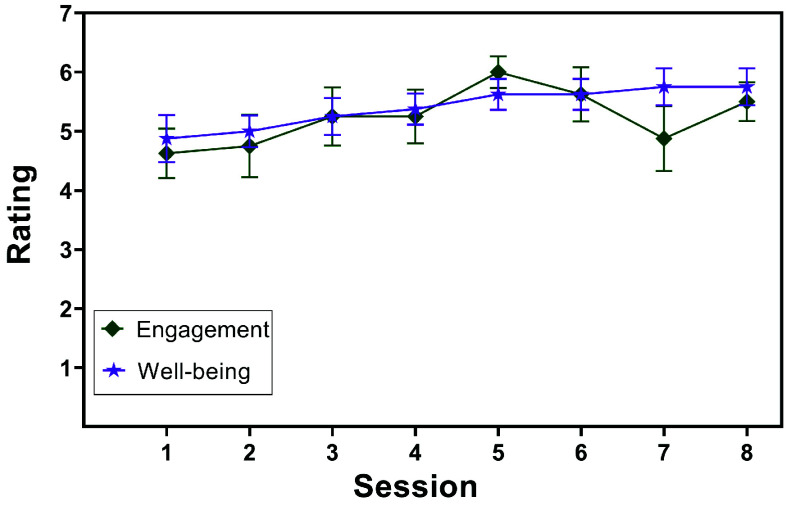
Post-session parent ratings of child-GoBot engagement levels and usefulness for child well-being per session. The markers show the mean, and the error bars illustrate the standard error.

*Survey Results:* Responses to the engagement and well-being questions from the post-session survey appear in Fig. 11. The data demonstrates the tendency for the mean engagement and well-being ratings to increase over time. The standard error values are small, which signifies a small spread in the ratings across the participant group.

For the three components of the NARS questionnaire (i.e., interaction, social, and emotional aspects of human-robot interaction) there was no significant change in the interaction ( $p=0.138$; $M=2.312$, $SD=1.151 $ before; $M=2.480$, $SD=1.337$ after), social ( $p=0.685$; $M=3.225$, $SD=1.493$ before; $M=3.175$, $SD=1.534$ after), or emotion ( $p=0.075$; $M=3.125$, $SD=1.227$ before; $M=2.583$, $SD=1.381$ after) ratings between the start and end of the experiment. There was no significant difference between trust ratings at the start and end of the study ( $p=0.606$; $M=71.375$, $SD=27.130$ before; $M=73.750$, $SD=29.698$ after).

## VI. Discussion

The aim of this research was to investigate how children respond to GoBot and assess its effectiveness in promoting physical movement over a longitudinal study.

Our results show support for **H1**; GoBot tended to promote more movement in both experimental conditions (i.e., teleoperated and semi-autonomous) compared to the control condition. This result was previously hinted by the ankle movement count and was better supported by the results from the normalized overground movement. The normalized overground movement data bolster the ankle movement data by resulting in similar outcomes with a less risky technique (actual child distance traveled, rather than just vigorous movements of the ankle. Further, the difference in normalized overground movement was significant between the control and semi-autonomous conditions. The trend of more motion during interactions with an active robot remained consistent over time. Across longitudinal sessions, only the results from the second session’s teleoperated condition fell below the baseline levels for both child movement and ankle movement. Anecdotally, parent free-response input, the trust ratings, and most NARS results corroborate the notion of the robot’s positive impact. Related parent statements included “robots encourage [...] interaction [and] make children excited to play,” and the tendency for a higher trust rating and lower social and emotional scale means for the NARS after the study (i.e., less concern that a robot would be a bad influence on children and feeling more comfortable being with the robot) are promising. These results tentatively support the idea of implementing robots to encourage physical movement to improve the health of (and healthcare support for) children. For example, these robots could be implemented in early physical therapy or early intervention settings, or as part of early childcare infrastructure (similar to current support from the USDA for healthy food in daycare settings).

The results provide support for **H2**. There were no significant differences between either motion level outcome for the teleoperated and semi-autonomous conditions when analyzing the ankle movement count and the normalized child movement. This outcome is encouraging as it suggests that semi-autonomous robot behaviors, which may be more easy-to-use and practical in intervention settings, can be equally effective as more effort-intensive direct teleoperation. As one parent pointed out, this means that (as one example) a semi-autonomous robot could potentially “keep children active even when [a parent] might not be able to entertain [their child].”

Our results, from the ankle movement count and normalized child movement, did not support **H3**. Contrary to our initial hypothesis, in almost all cases, child movement remained consistently higher than baseline throughout the entire study. Parent ratings, which showed that the robot’s perceived abilities to engage and enhance well-being tended to improve over time, support this idea that the robot remained effective. We found this lack of movement and engagement drop-off, especially in a population with such a short attention span, to be encouraging. Compared to other types of toys and electronic devices, robots may possess added potential for long-term intervention success which may enhance healthcare for children in ways that were not previously possible.

The results support **H4**; there was no significant difference between the child’s responses to the teleoperation and semi-autonomous condition. For both conditions, the children maintained a similar average distance rate from the robot. This result is promising because it further supports the conclusion that semi-autonomous robot behaviors can be equally effective to effort-intensive teleoperated behavior

*Key strengths* of this research include its relatively long-term duration and its within-subjects design as recommended by primers for best human-robot interaction research practices such as [43]. Within-subject design can help account for individual differences, which can be formidable. Our advantageous design elements enable us to gain insights into the robot’s impact beyond the initial novelty effect and helped minimize the noise in our data and display how traces for individuals match the aggregate trend. Additionally, the assessment of the system with young children is noteworthy in the field of assistive robotics. It is uncommon to find human-robot interaction research with users below three years of age, such as the participants in our study.

Our study also involves certain *limitations*. We encountered typical challenges associated with working with young children, including fluctuations in mood during the sessions and variations in individual children’s interests. Additionally, the interaction times during the study were relatively short, consisting of five-to-ten-minute conditions.This paper expands our insights from a past preliminary publication on the same study topic, so although the current work presents more robust findings and new self-report-based insights, it was not possible to collect further experimental data. To overcome these limitations, future endeavors could benefit from a larger sample size, additional study sessions, and longer interaction periods.

## VII. Conclusion

The presented study spanned two months and evaluated the effects of three conditions (two experimental, one baseline) on child motion. The results consistently indicated that active robot interventions during play sessions tended to promote more physical activity, perhaps via the mechanism of encouraging children to approach the robot. Furthermore, the trend in motion levels persisted throughout all of the relatively long duration of the two-month study. Overall, this work highlights the potential of assistive robots to influence child physical activity. The similarity between the results for the teleoperated and semi-autonomous conditions suggests that users of this type of robotic system could potentially conserve direct human effort and allocate these resources towards more enriching interaction efforts without compromising the success of motor interventions. Researchers in the fields of robotics and child motor interventions stand to benefit from the insights garnered through this study.
